# Electrophysiological Characteristics of Dorsal Raphe Nucleus in Tail Suspension Test

**DOI:** 10.3389/fnbeh.2022.893465

**Published:** 2022-05-31

**Authors:** Liuchang Zhou, Dan Liu, Zedan Xie, Di Deng, Guoqi Shi, Jinlan Zhao, Shasha Bai, Lei Yang, Rong Zhang, Yafei Shi

**Affiliations:** ^1^School of Fundamental Medical Science, Guangzhou University of Chinese Medicine, Guangzhou, China; ^2^Joint Laboratory for Translational Cancer Research of Chinese Medicine of the Ministry of Education, International Institute for Translational Chinese Medicine, School of Pharmaceutical Science, Guangzhou University of Chinese Medicine, Guangzhou, China; ^3^School of Chemical Biology and Biotechnology, Peking University Shenzhen Graduate School, Shenzhen, China; ^4^School of Foreign Studies, Guangzhou University of Chinese Medicine, Guangzhou, China

**Keywords:** *in vivo* electrophysiology, tail suspension test, dorsal raphe nucleus, serotonergic (5-HT) nuclei, despair-like behavior

## Abstract

The dorsal raphe nucleus (DRN) is a major source of serotonin in the central nervous system, which is closely related to depression-like behaviors and is modulated by local GABAergic interneurons. Although serotonin neurons are known to be activated by struggling behavior in tail suspension test (TST), the exact electrophysiological characteristics are still unclear. Here, we combined *in vivo* electrode recording and behavioral test to explore the mice neuron electrophysiology in DRN during TST and observed that gamma oscillation was related to despair-like behaviors whereas burst fraction was crucial for survival-like behaviors. We reported the identification of a subpopulation of DRN neurons which change their firing rates when mice get into and during TST immobile states. Both increase (putative despair units, D units for short) and decrease (putative survival units, S units for short) in firing rate were observed. Furthermore, using optogenetics to identify parvalbumin-positive (PV+) and serotonin transporter-positive (SERT+) neurons, we found that SERT+ neurons were almost S units. Interestingly, those that have been identified PV+ neurons include ~20% of D units and ~50% of S units. These results suggest that electrophysiological characteristics incorporated in despair-like behavior studies can provide new insight into the study of anti-depression targets, and GABAergic interneuron is a complex key hub to the coding and regulation of local neural network.

## Introduction

As a crucial region for serotonergic system, dorsal raphe nucleus (DRN) contains the largest population of serotonergic (5-HT) neurons in the brain (Descarries et al., 1982). Clinical antidepressants, such as selective serotonin reuptake inhibitors (SSRIs), mainly target on the reduction of 5-HT turnover in the DRN 5-HT system. Dysfunction of the serotonergic system is implicated in depression-like behaviors. As a general balancing mechanism, ventral and lateral wings of GABAergic inputs play a key role in the modulation of DRN 5-HT neurons (Challis et al., 2013; Li et al., 2016, 2020). They are the most prevalent type of non-5-HT neurons in DRN, representing about 25–30% of the total population (Johnson, 1994; Hernandez-Vazquez et al., 2019).

However, the delicate functional organization of DRN remains unclear in molecular level, especially in electrophysiology. To decode the depression-like behaviors in central nervous system, a series of studies have been focusing on the immobility behavior of tail suspension test (TST) modulating from DRN. Early research found that prefrontal cortex-DRN projection controls the response of behavioral challenge (Warden et al., 2012), which arouses interests of exploring the coding of depression-like behaviors. A calcium imaging research reconfirmed an increase in DRN 5-HT fluorescence at the onset of movement in TST (Seo et al., 2019). Although all these studies brought hope to the precise regulation and therapy of depression-like behaviors, two possible limitations are 5-HT neuronal heterogeneity and calcium imaging resolution.

*In vivo* electrophysiology single-unit recording provides a new dimension in this field. The relationship between depression-like behavior and electrophysiology heterogeneity of 5-HT neurons in DRN remains unknown. Although 5-HT neurons are traditionally considered to be homogeneous, they are different both in molecular identity and in electrophysiological property (Cohen et al., 2015; Okaty et al., 2015, 2020; Fernandez et al., 2016; Commons, 2020), which suggests that there exist distinct functional subtypes in behavioral coding. Moreover, electrophysiology signal recording at first hand has higher resolution than calcium imaging recording that converted to fluorescence signal.

Here, we explored electrophysiological characteristics of DRN in TST by combining our previous *in vivo* opto-electrophysiology (Deng et al., 2020a,b; Xia et al., 2022) and behavioral test. We first found gamma oscillation (25–40 Hz) in local field potential, and nearly 70% neurons in single unit recorded in DRN were related to immobile states of TST. So far, this experiment provides a new potential electrophysiology index for the immobility identification in TST. To further explore the relationship between electrophysiology heterogeneity and behavior coding, we then identified and calculated TST immobility-related serotonin transporter-positive (SERT+) and parvalbumin-positive (PV+) neurons in freely behaving mice with implanted electrodes and opto-fiber to Cre-label mice. In the DRN, the firing of almost all related units of SERT+ neurons increased when mice were in mobile states in TST, it meant seeking for survival, and this group was called putative survival unit (S unit). Few (~3%) SERT+ neurons firing increased when mice were in immobile states in TST, it meant giving up struggling, and this group was called putative despair unit (D unit). As for PV+ neurons, there are ~50 and ~20% of putative survival and putative despair units, respectively. Interestingly, the burst firing pattern of SERT+ neuron was crucial for survival behaviors. We reported SERT+ and PV+ neurons electrophysiological characteristics of DRN in TST, and burst firing pattern indicated a potential drug target for reversing despair-like behaviors.

## Methods

### Animals

SERT-Cre mice (Zhuang et al., 2005; strain name B6.Cg-Tg(Slc6a4-Cre)ET33Gsat, Mutant Mouse Resource and Research Center, USA, RRID: MGI_3691580) and PV-Cre mice (Hippenmeyer et al., 2005) (B6;129P2-Pvalbtm1(Cre) Arbr/J, Jackson Laboratory, stock no. 008069) were originally provided by Dr. Minmin Luo [National Institute of Biological Sciences (NIBS), China] and Dr. Weihai Chen (Southwest University), respectively, and then further bred in the laboratory. PV-Cre and SERT-Cre mice were group housed (5–6 mice/cage) under a 12-h light/dark cycle (8:00 AM−20:00 PM) and were provided with food and water *ad libitum*. The 8-week-old male mice were used in this study.

For *in vivo* electrophysiology experiments, mice were singly housed after the surgery that implanted multi-channel electrodes. All animal experiments were performed in accordance with the ARRIVE (Animal Research: Reporting of *in vivo* Experiments) guidelines, approved by the Ethics Committee of Guangzhou University of Chinese Medicine.

### 16-Channel Opto-Fiber-Electrode Making

The 16-channel opto-fiber-electrode (opto-electrode) was composed of an optical fiber (O.D. 200 μm, length 6.0 mm, FOC-C-1.25-200-0.37-6.0, Inper Inc., China) and multi-wire electrodes (the end of optic fiber is ~200–300 μm above the tips of the recording electrodes, which was handmade on printed circuit board [PCB, Top-Bright Inc., Tb-OA16, thickness:200 μm, space between connecting finger:1,270 μm)] with optical fiber jack, and was soldered to female header connector. The optical fiber was glued to the jack with 495 instant adhesives. The electrode array consisted of 16 individually insulated nichrome wires (35-μm inner diameter, impedance 300–900 Kohm; Stablohm 675, California Fine Wire). Arrays of 16 wires were arranged in a 3 × 5 × 5 × 3 pattern with a reference electrode on the top and stabilized with jewelry gel (Pacer Technology Inc., ZAP, PT-27).

### Virus Injection and Multi-Wire Electrode Implantation

Mice were anesthetized with isoflurane (~1% in a gas mixture) in a stereotaxic apparatus (RWD Life Science Co., China). Viruses [PT-0002, rAAV2/9-EF1α-DIO-hChR2(H134R)-mCherry-WPRE-hGH, BrainVTA Wuhan, China] were slowly (300 nl, 50 nl/min) injected to DRN (−4.36 mm posterior to bregma, 1 mm lateral to midline) with a glass pipette (504949, WPI) controlled by a microsyringe pump (788130, kd scientific Inc., Holliston, MA, USA), which was operated by a 22° entry angle toward the midline and 2.70 mm ventral from cortical surface through the left hemisphere to avoid puncturing the vessel and the fourth ventricle. The glass pipette was left in place for extra 10 min and then slowly withdrawn. Following injection, the opto-electrode was implanted slowly as described previously (Deng et al., 2020b). The implanted electrodes were secured with dental cement. After surgery, the mice were habituated individually for 10–20 days for recovery and ChR2 expression.

### Opto-Genetics and *in vivo* Recording

Mice were trained to be habituated to handling and recording procedure before behavioral test. During home cage and tail suspension test, the external portion of the chronically implantable cannula and electrode female header were coupled with an optical fiber and an electrode preamplifier, respectively. Broadband (0.3 Hz−7.5 kHz) neural signals were simultaneously recorded (16 bits ^@^ 30 kHz) from implanted 16-channel arrays using a 32-channel data acquisition system (Apollo, Bio-Signal Technologies, McKinney, TX).

In some previous studies, we analyzed waveform and electrophysiological characteristics to identify PV/fast-spiking-neurons (Yao et al., 2019; Deng et al., 2020b). To be more convincing, both optogenetic stimulation and waveform/electrophysiological characteristics were used in this study to identify the PV neurons and SERT neurons. Therefore, after 10-min TST, a blue light pulse (465 nm, 2 min, 20-ms pulse width, 20 Hz, ~10 mW at end of optic fibers) was applied with B1-465 (Inper Inc., China) to identify the type of neurons. Neurons were considered as PV or SERT types when they showed a significant increase in their neuronal activity upon 465-nm illumination.

### Tail Suspension Test

During the TST, tail of the mouse was stuck on strips tape. To ensure that mice could not touch or climb during the test, the end of strip tape was then secured to a horizontal bar 40 cm from the ground. In the electrophysiological recording experiment, after about 10 min of free exploration in their home cage, SERT/PV-Cre mice were moved to TST for 10 min. During the TST, only electrophysiological recording was conducted, without 465-nm light illumination. Video recording started when the mice were inverted and taped for 10 min. The time of their struggling was measured by blind scoring of video material after the test was completed.

### Data and Statistical Analysis

After recording, the waveform of extracellular action potentials of all neurons recorded by each channel was aligned and sorted according to the principal component analysis (PCA) cluster in a three-dimensional principal component space using an offline-sorter software (Plexon) as described before (Deng et al., 2020b). Waveform, firing rate, inter-spike interval, peri-event histograms, power spectrum densities, and oscillation were analyzed with Neuro Explorer V5 (Plexon). Putative despair units and putative survival units represent DRN neurons that increase or decrease their firing rates when mice during the TST immobile state. These changes were evident whenever transitions occur from TST mobile state to immobile state. So, we defined subpopulations of DRN neurons that change their firing rates when mice at the moment of transition between behavioral response types and sustained differential firing rates throughout the duration.

The number of mice used (*N*) and neurons recorded (*n*) in this experiment were shown in the figure legends. Statistical significance was calculated using two-tailed paired/unpaired *t*-test, one-way/two-way RM ANOVA (version 8, GraphPad Prism software, USA), as noted. Data were reported as mean ± SEM. Significance levels were noted as * (*p* < 0.05), ** (*p* < 0.01), *** (*p* < 0.001), **** (*p* < 0.0001).

### Perfusion and Location Confirmation

At the end of an *in vivo* recording experiment, recording sites were marked by passing 10 s 100 μA current through each electrode with Lesion Making Device (53500, Ugo Basile, Italy). Electrode locations were confirmed using immunohistology on the co-localization of viral fluorescence. Mice were perfused with phosphate-buffered saline (PBS, 0.1M, pH 7.4, R160015, REBIO, Shanghai, China) followed by 4% paraformaldehyde (PFA, 158127, Sigma). After perfusion, the brains were post-fixed for 24 h in PFA, then were cryoprotected with 20% sucrose in PBS at 4°C until sank, and then transferred to 30% sucrose in PBS until sank. Brains were embedded in optical cutting temperature (OCT, Tissue-Tek 4583, Sakura Finetek Inc., USA) and cut into 30-μm frozen sections with cryostats (Leica CM1860 UV, Germany). The electrode locations were reconstructed DAPI (ab228549, Abcam) staining and imaging on a confocal microscope (Leica SP8, Germany). The distribution of serotonergic neurons in DRN was observed by immunofluorescence. Mice' brain slices were incubated with blocking solution (10% normal goat serum and 0.3% Triton X-100 in PBS) and then incubated 1 h at room temperature. Primary antibody rabbit anti-Tph2 (1:500; ab184505; Abcam) was diluted in blocking solution and applied overnight at 4°C. The next day, brain slices were washed three times with PBS followed by secondary antibody goat anti-rabbit IgG (1:500; ab150077; Abcam) for 2 h at room temperature. Slices were then washed three times in PBS. Experimental procedures of nuclear staining with DAPI and confocal photography were the same as previously described.

## Results

### Changes in Firing Rate in DRN Neurons Associated With Changes in TST States

**Figure 1:**
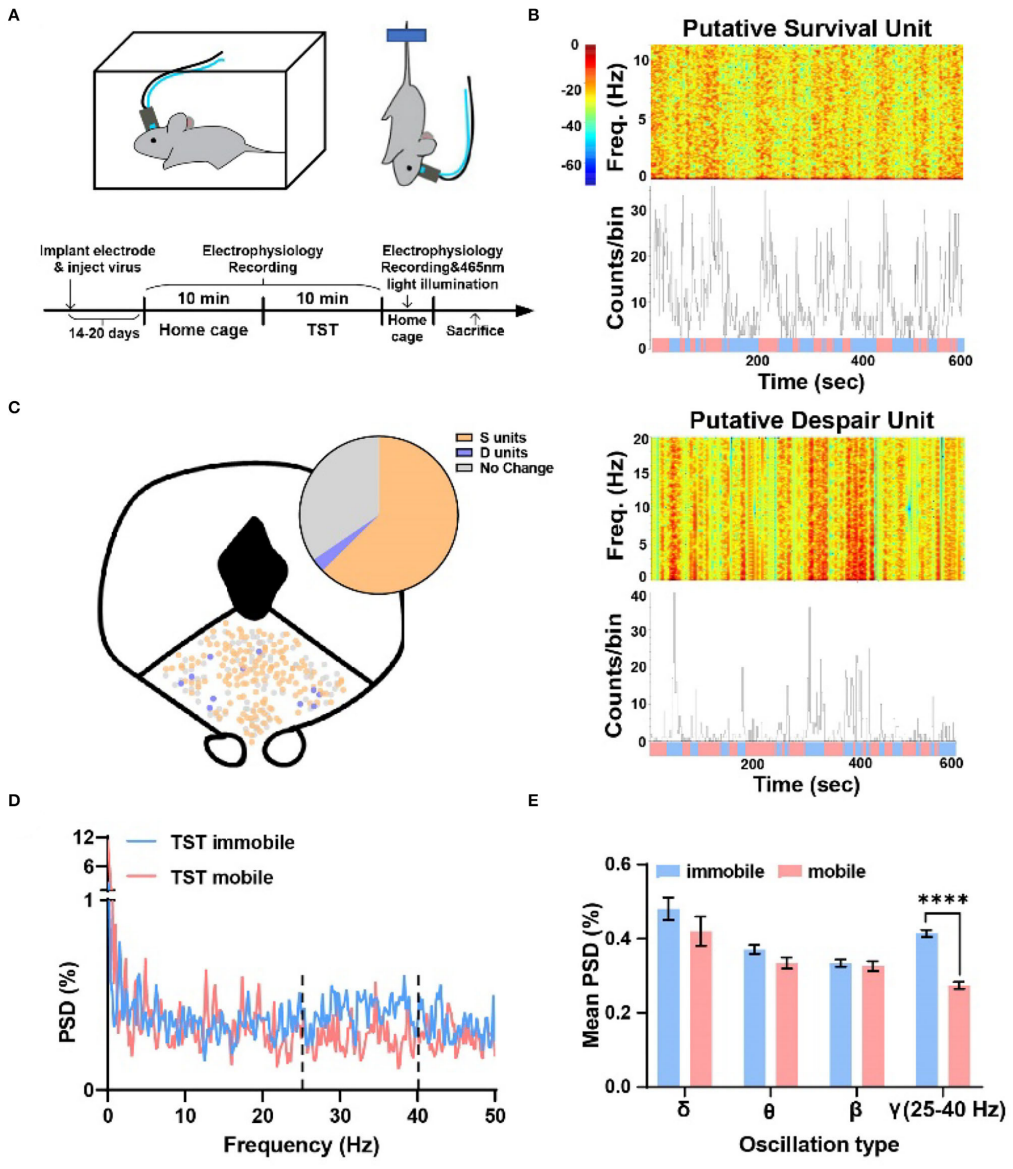
Two types of units showing opposite changes during different states transition in TST, and the local field potential gamma oscillation of DRN anticipated despair-like behavior. **(A)** (Top) Behavioral test assembly. (Bottom) Experimental procedure. **(B)** (Top) Sample recording of a putative survival unit (S unit) during TST. Spectrogram and rate histogram showing drastic reduction in spike rate during the transition from TST mobile to immobile. (Bottom) Sample recording of a putative despair unit (D unit) during TST. Spectrogram and rate histogram showing a remarkable increase in spike rate during the transition from TST mobile to immobile (blue means immobile states, and red means mobile states). **(C)** The spatial location of different types of units in DRN during the transition from mobile to immobile in TST. *N* = 21 mice, total units = 248, S units = 150 (~61%), D units = 11 (~4%). **(D,E)** Sample of the power spectral densities (%) of TST mobile and immobile in DRN. The mean power spectral densities (%) during TST mobile and immobile states in different oscillation types. The mean PSD (%) of gamma oscillation (25–40 Hz) showing a clear increase during immobile states (two-tailed paired *t*-test, ****p < 0.0001).

To examine whether DRN neurons were associated with different TST states, neural activity was recorded by using a 16-channel opto-electrode array targeted to DRN, and two epochs of data were recorded: a 10-min epoch in the home cage and a 10-min epoch during the TST (Figure 1A). There was a clear change in firing rate in part of neurons recorded when mice changed their states from mobile to immobile in TST and the firing rates of them showed either decrease or increase (Figure 1B). It was shown that about 61% of DRN neurons decreased their firing rate from TST mobile to immobile states, while about 4% increased (Figure 1C top, S units = 150, 60.5%; D units = 11, 4.4%). There was no obvious location difference in different types of units (Figure 1C bottom). Thus, two distinct populations of DRN neurons were found, which showed opposite changes in their firing rates during TST states changing, and suggested that these DRN neurons could code state representing survival or despair-like behaviors.

### Coding of Despair-Like Behaviors Was Related to the Gamma Oscillation

To understand whether oscillation of DRN neurons codes despair-like behaviors, an analysis of the percent of power spectral densities (PSD%) of TST mobile and immobile states in DRN was conducted. It was found that the PSD% of TST immobile state was higher than mobile state between 25–40 Hz (Figure 1D). Further analysis indicated that gamma oscillation (25–40 Hz) showed significant enhancement during TST immobile states (Figure 1E). The results indicated that gamma oscillation related to despair-like behaviors.

### Majority of SERT-Positive Neurons in DRN Belonged to Putative Survival Unit

**Figure 2:**
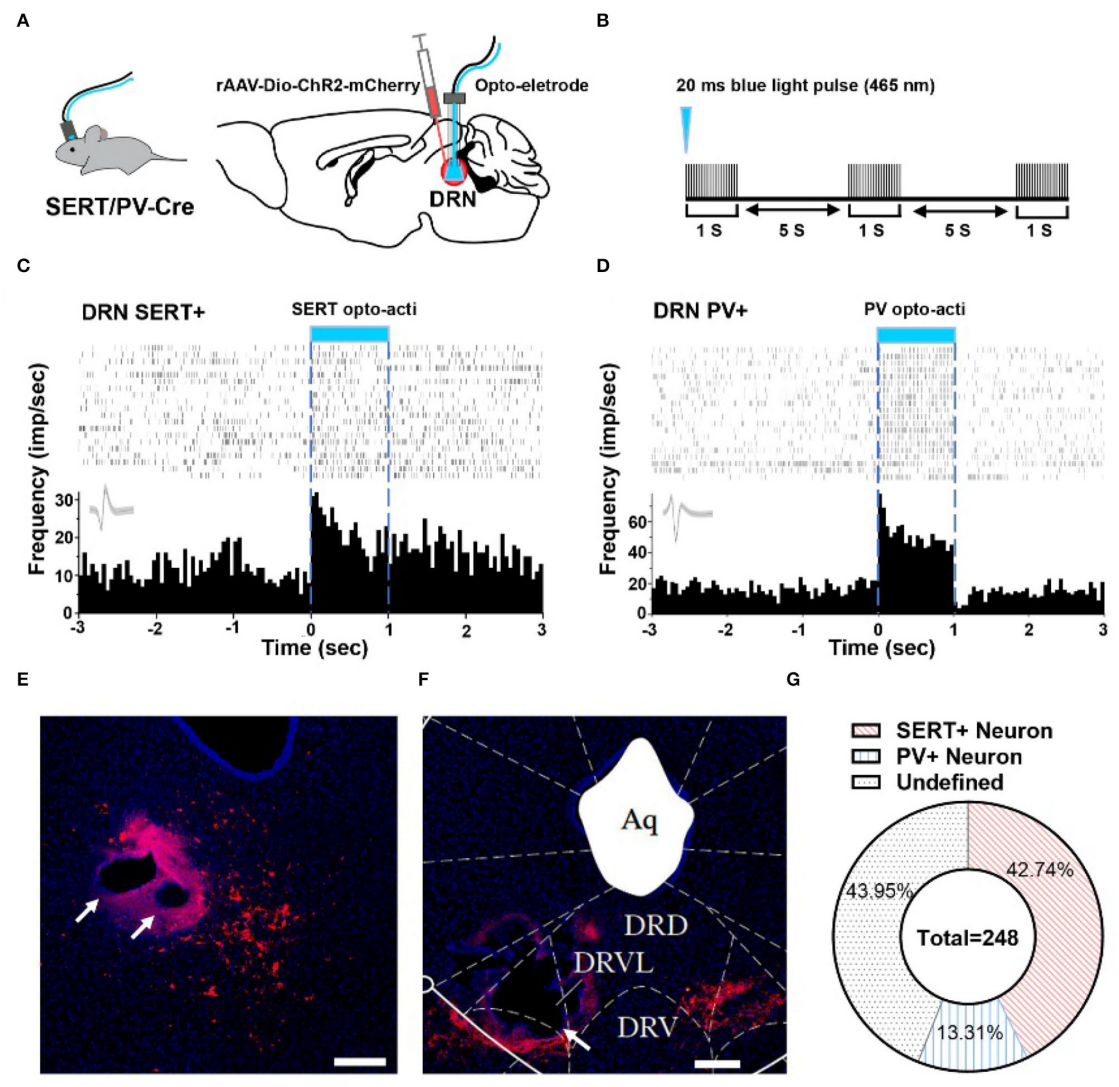
Optogenetic identification of DRN SERT+ and PV+ neurons. **(A)** Left: SERT/PV-Cre mice with opto-electrode assembly. Right: injection and stimulation configuration. **(B)** Using opto to distinguish SERT/PV neurons, Opto tag: 20 ms, 20 Hz, 10 mW. **(C)** Optogenetic identification of DRN SERT+ neurons based on their responses to optogenetic activation using optrode. **(D)** Response of PV+ neurons during optogenetic activation of DRN PV+ neurons. **(E)** Sample image shows the injection site of DIO-ChR2-mCherry and opto-electrode locations in DRN of SERT-Cre mice. Bar, 200 μm. **(F)** Sample image shows the injection site of DIO-ChR2-mCherry and opto-electrode locations in DRN of PV-Cre mice. Bar, 200 μm. **(G)** Proportion of different types of neurons recorded in DRN (SERT-Cre mice = 13, 5-HT neurons = 106; PV-Cre mice = 8, PV neurons = 33; undefined neurons = 109).

To explore whether 5-HT neurons and PV neurons in DRN corresponded to putative survival units and putative despair units, respectively, we used optogenetic activation to identify them and combined them with behavioral test. By injecting rAAV-DIO-ChR2-mCherry in SERT-Cre or PV-Cre mice, we can label and manipulate 5-HT/PV neurons with 465 nm blue light delivery (Figures 2A,B). Identification of 5-HT/PV-positive neurons was based on their significant time-locked increase in firing frequency upon blue light illumination (Figures 2C,D).

In this study, a total of 248 units in 21 mice were recorded, on average 12 neurons per mouse. To confirm whether recording position is DRN or not, we adopted electrolytic lesion and staining after recording (Figures 2E,F). After identifying the neuron type by optogenetic and confirming implant position, it showed that 42.74% SERT+ neurons and 13.31% PV+ neurons were recorded in DRN (Figure 2G).

**Figure 3:**
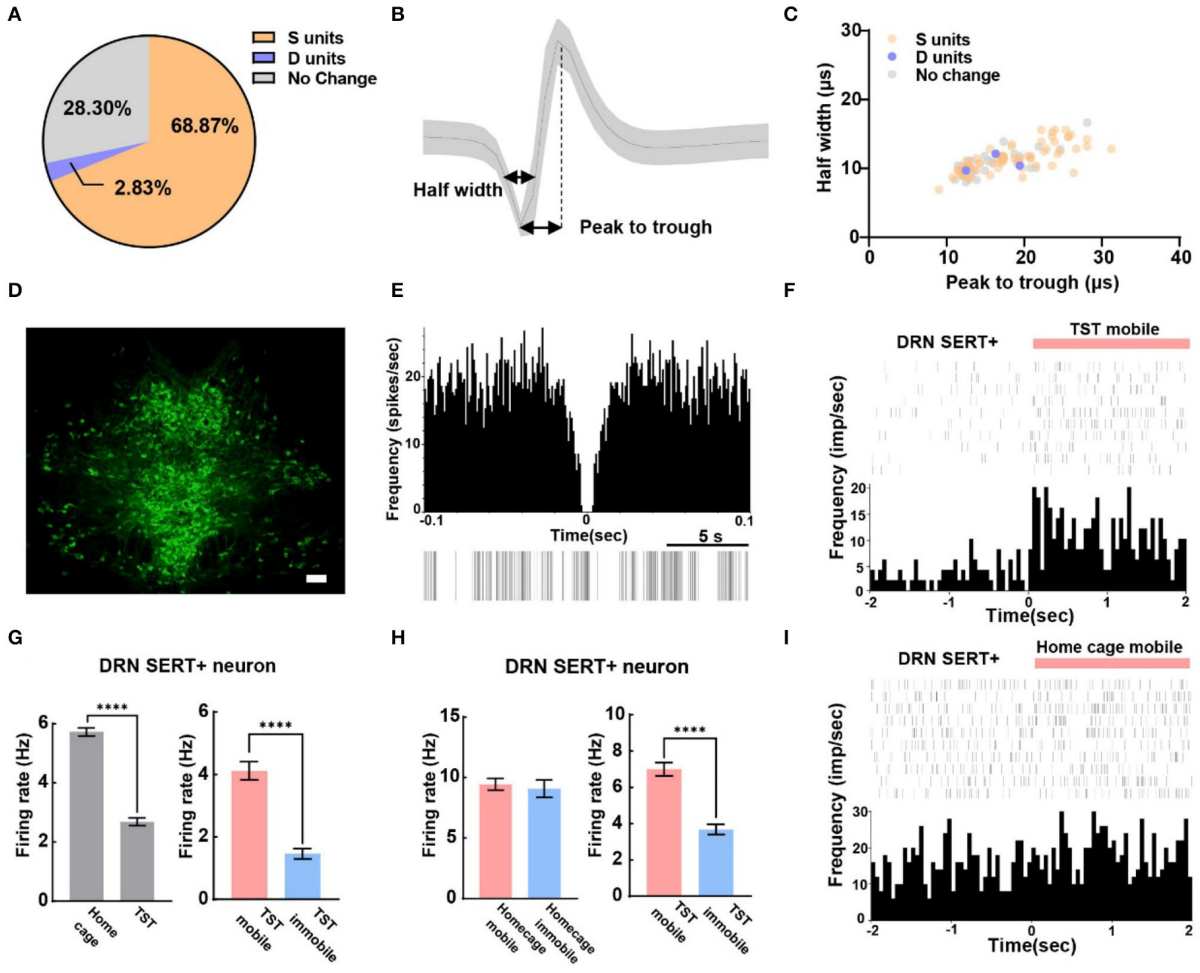
The majority of serotonergic neurons in DRN are putative survival units. **(A)** Proportion of different types of units recorded in DRN SERT+ neurons. *N* = 13, *n* = 106, S units = 73, D units = 3. **(B)** Example of measuring the half width and peak to trough of a waveform. **(C)** Scatterplot of different units based on their spike waveform of DRN SERT+ neurons which identified by optogenetics. **(D)** Representative photomicrographs (10X) of TPH2 antibody-stained serotonin neurons in DRN. Bar, 100 μm. **(E)** The autocorrelogram, waveform, and the raster of a single SERT+ neuron recorded in this study, respectively. **(F)** Spike frequency of an example DRN SERT+ neuron from the TST immobile to mobile stages. **(G)** Bar pot of DRN serotonergic neuron that is inhibited during TST (left), especially in immobile states (right). **(H)** Bar pot of serotonergic neuron that is only inhibited in TST immobile states (right) not home cage immobile (left). **(I)** Spike frequency of an example DRN SERT+ neuron from home cage immobile to mobile stages (two-tailed unpaired *t*-test, ****p < 0.0001).

A total of 106 SERT+ neurons were recorded, among which 68.87% of them were S units and 2.83% were D units (Figure 3A). To explore whether waveform characteristics of SERT+ neurons coding despair-like behaviors, we measured the half width and peak to trough of waveform of these units (Figure 3B). Cluster analysis indicated that waveform heterogeneity of SERT+ neurons was independent of correlation to TST behavior coding (Figure 3C). The distribution of 5-HT-positive neurons in the DRN is concentrated in the midline region according to the immunofluorescent staining (Figure 3D). The characteristics of the spontaneous spike activities of SERT+ neurons in the DRN recorded in this study are illustrated in Figure 3E. In addition, we observed that activities of most DRN SERT+ neurons changed during behavioral test in a way which may specifically reflect the decision to mobile or immobile states during the TST (Figure 3F). This neuron was highly active during the home cage stage (Figure 3G left), but during the TST, it stayed active during mobile states and was inhibited during immobile states (Figure 3G right). To explore whether neurons exhibited similar phenomenon during home cage stage, we also analyzed SERT+ neuronal activity during mobile and immobile states in home cage. The neuronal firing rate was no significant difference in switching from mobile to immobile states in home cage (Figure 3H left), but decreased in TST (Figure 3H right). The frequency histogram in home cage showed the same result (Figure 3I).

### PV-Positive Neurons May Bidirectional Regulate Despair-Like Behaviors

**Figure 4:**
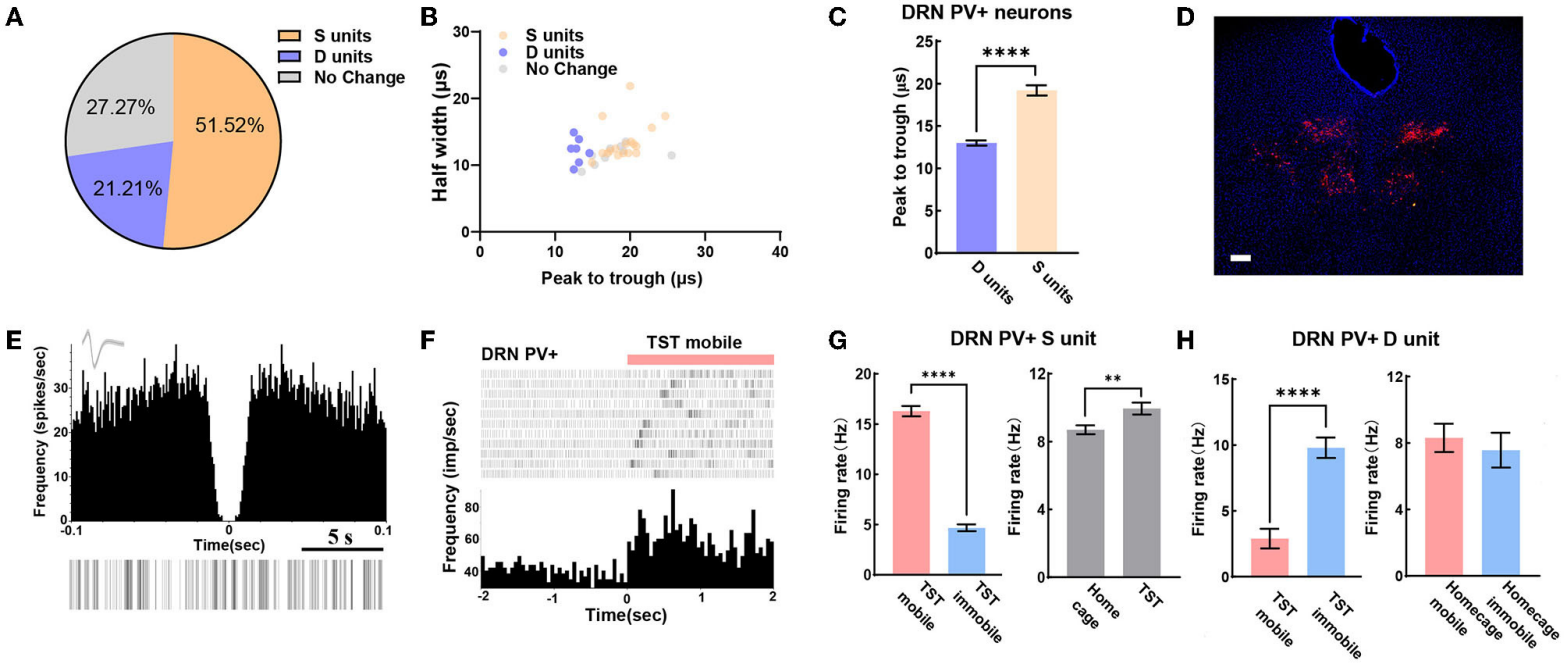
DRN PV+ neuronal activity may bidirectional regulate despair-like behaviors. **(A)** Proportion of different types of units recorded in DRN PV+ neurons. *N* = 8, *n* = 33, D units = 7, 21.21%; S units = 17, 51.52%. **(B)** Scatterplot of different units based on their spike waveform of DRN PV+ neurons which were identified by optogenetics. **(C)** Bar pot of peak to trough of putative survival units and despair units of DRN PV+ neurons. **(D)** Sample image showed the injection site of Dio-ChR2-mCherry locations in DRN of PV-Cre mice. Bar, 200 μm. **(E)** The autocorrelogram, waveform, and raster of a single PV+ neuron recorded in this study, respectively. **(F)** Spike frequency of an example PV+ neuron in DRN from TST immobile to mobile stages. **(G)** Bar pot of PV+ S units in DRN that is activated during TST (right), especially in mobile states (left). **(H)** Bar pot of PV+ D units in DRN that is activated during immobile state in TST (left), but not in home cage (right) (two-tailed unpaired *t*-test, **p < 0.01, ****p < 0.0001).

We can also reliably identify PV+ neurons in DRN by opto-electrode recording method. In the experiment, a total of 33 PV+ neurons in DRN from 7 PV-Cre mice were recorded, including 21.2% of D units and 51.5% of S units (Figure 4A). Meanwhile, we found that peak to trough of waveform of D units was shorter than that of S units (Figures 4B,C). The confocal image showed that very few PV+ neurons were found in the midline of DRN (Figure 4D), which was different from the distribution of SERT+ neurons (Figures 2E, 3D). The characteristics of the spontaneous spike activities of PV+ neurons in the DRN recorded in this study are illustrated in Figure 4E. Previous classical studies have shown that PV+ neuron activity is opposite to serotonergic neuron, which means PV+ neuron in DRN region may be activated during immobile states in TST (Zhou et al., 2017). Nevertheless, some PV+ neurons recorded in our experiment had lower firing rate during TST immobile states than mobile states (Figures 4F,G left). However, the firing rates of these PV+ neurons had an elevation in TST compared to home cage (Figure 4G right), which was different from SERT+ S units (Figure 3G left). Interestingly, the firing rates of PV+ D units strongly increased while mice switched from mobile to immobile state in TST but not at home cage (Figure 4H). These results indicate that PV+ neurons in DRN region may bidirectionally regulate despair-like behaviors.

### TST Immobile States Had Fewer Burst Spikes

**Figure 5:**
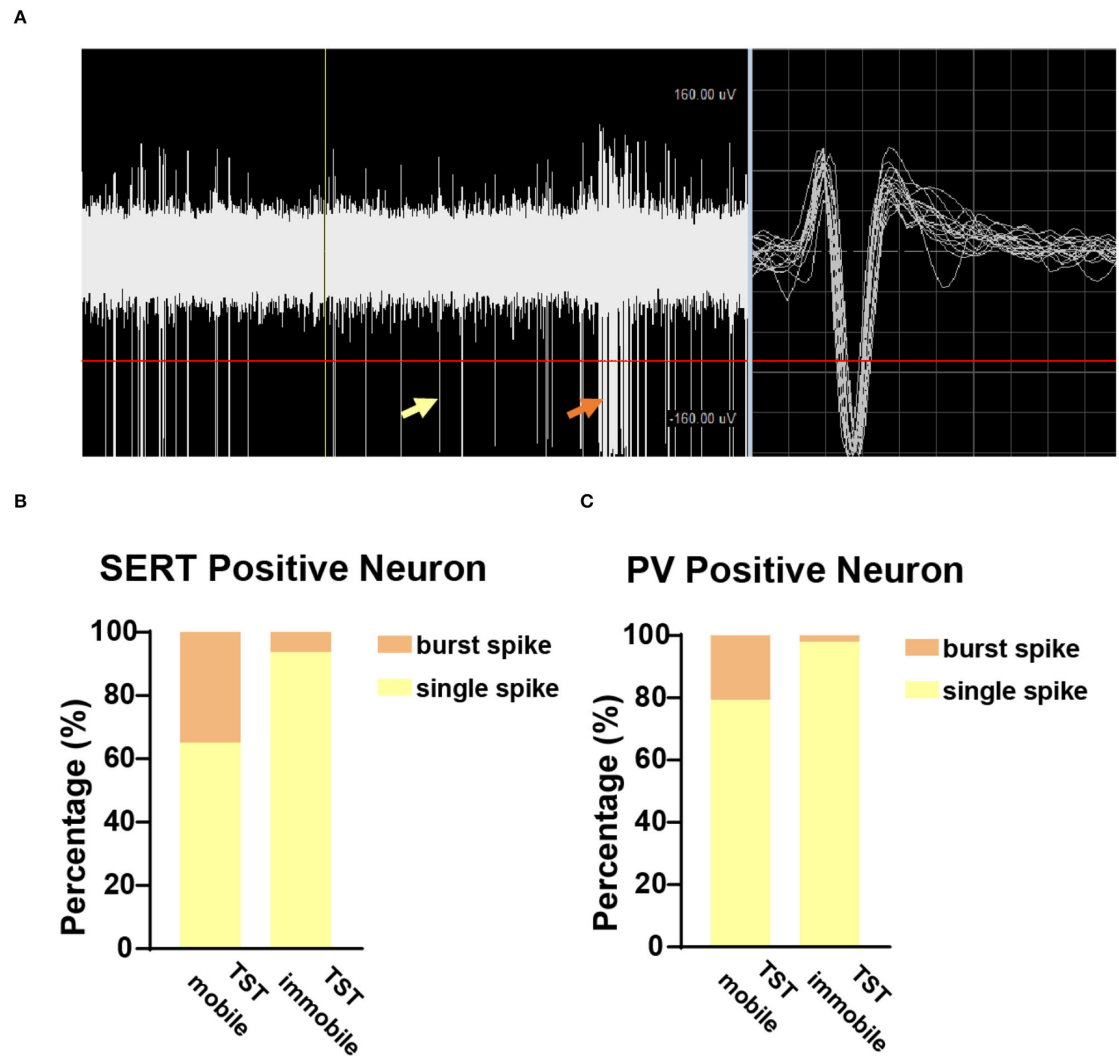
Few burst spikes during the immobile state in TST. **(A)** Sample picture showed burst spike (orange arrow) and single spike (yellow arrow) while recording. **(B)** The proportion of burst and single spike in SERT^DRN^ neuron firing during TST mobile and immobile states. **(C)** The proportion of burst and single spike in PV^DRN^ neuron firing during TST mobile and immobile states.

To further explore the characteristics of neuronal firing types in TST mobile and immobile states, we analyzed the ratio of burst spikes and single spikes in different states of TST. The burst spike and single spike are indicated by orange arrow and yellow arrow, respectively, in the recording sample (Figure 5A), and the TST immobile states have fewer burst spikes compared with single spike, whether it is SERT+ (Figure 5B) or PV+ (Figure 5C) neuron. These results indicate that there are fewer burst spikes in DRN corresponding to despair-like behaviors (TST immobile states).

## Discussion

Although many studies tried to decode depression/defensive/reward/threat behaviors in DRN by calcium imaging method (Warden et al., 2012; Li et al., 2016; Huang et al., 2017; Seo et al., 2019), few studies focused on TST behavior coding by combining *in vivo* electrophysiology with behavioral test, so that the percentage and features of these related neurons remained unclear. To clarify the role of DRN in the encoding of despair or survival-like behavior, we recorded *in vivo* spiking activities of DRN neurons during the TST. We found that gamma oscillation and burst fraction were the crucial electrophysiological characteristics in TST behavior coding. We further applied optogenetics to identify two types of neurons and it showed the following differences: (1) Most of SERT+ neurons were putative survival units. (2) PV+ neurons showed bidirectional changes during despair-like behavior. (3) Waveform heterogeneity of SERT+ neurons was irrelevant to TST behavior coding, whereas PV+ putative despair unit was narrower in peak to trough of waveform. In addition, our results suggest that coding of despair-like behaviors in DRN is likely mediated by the gamma oscillation, while survival behaviors mediated by burst fraction, because there is significant gamma oscillation and fewer bursting spikes during the immobile phenotype in TST.

The relationship between gamma oscillation and despair (one of the depression-like behaviors) is still not clear. Antidepressants sometimes have opposite effects on gamma oscillations, with 5-HT agonist-suppressing and NE agonist-enhancing gamma oscillations (Hajos et al., 2003; Akhmetshina et al., 2016; Fitzgerald and Watson, 2019). A neglected but crucial problem is that gamma power may change dynamically and vary as a function of exact brain region, behavior states, and degree of alertness state. Previous study reported that increased gamma oscillations were associated with increased immobility time during TST in sleep deprivation depression model (Ahmed et al., 2021). Furthermore, we explored local field potential in DRN during TST and first reported that gamma oscillation was related to despair-like behaviors (Figures 1D,E).

To understand the delicate electrophysiological mechanism in TST, we performed *in vivo* recording during behavioral test with optogenetics to identify SERT+ and PV+ neurons. Conventional view suggests that 5-HT neurons have varying spike shapes and a low firing rate, yet these criteria have recently been put into question (Cohen et al., 2015; Luo et al., 2015). Previous patch-clamp studies have shown spontaneous firing activity of 5-HT neurons at a rate between 0.3 and 5.81 Hz (Mlinar et al., 2016), while GABA interneurons at an average rate of 6.6 Hz in DRN (Challis et al., 2014). We found that the spontaneous spike frequency rate of SERT+ neurons was between 2 and 9 Hz, and that of PV+ neurons more than 8 Hz, a result consistent with our previous finding in forebrain cortex (Deng et al., 2020b). Furthermore, about 70% of neurons were found related to survival-like or despair-like behaviors in our present study (Figure 1C). Although calcium imaging study has reported that DRN 5-HT neurons' activities increased when mice struggled in TST (Warden et al., 2012; Seo et al., 2019), two possible limitations are the heterogeneity and calcium imaging sensitivity of 5-HT neurons.

With opto-electrode recording, we identified 106 SERT+ neurons (Figure 2G). Most of the behavior-related neurons are putative survival units (Figure 3A), which is consistent with previous optogenetic stimulation studies (Nishitani et al., 2019; Seo et al., 2019). Together, these results suggest that an increased 5-HT release is a key factor in reducing stress. We first analyzed mobile and immobile firing rates in home cage (Figure 3H left) to avoid motion-induced electric noise in TST survival-like behavior. Previous studies reported that movement onset in the open field test was associated with a robust activity reduction in 5-HT neurons (Seo et al., 2019); therefore, we used home cage context as a blank control. Furthermore, we measured the waveform parameter and found that the same electrophysiological characteristics may encode different behaviors, although a previous electrophysiology study classified them with spiking properties (Mlinar et al., 2016). Considering the diversity of serotonergic neurons in DRN (Calizo et al., 2011; Okaty et al., 2015, 2019), more methods should be taken to distinguish their subtypes.

Most of the GABAergic cells in DRN form local connections, which are capable of exerting tonic inhibition on DRN 5-HT neurons (Gervasoni et al., 2000; Zhou et al., 2017; Hernandez-Vazquez et al., 2019). Although a negative immunostaining for PV+ in the DRN of rodents has been reported (Celio, 1990), recent studies suggest that PV+ neuron, including DRN, is one of the most extensively studied GABAergic neurons (Nascimento et al., 2020), which are distinctive from the other subtypes of GABAergic neurons due to its high-frequency spikes and critical contributions to gamma oscillation (Bartos et al., 2007; Sohal et al., 2009; Buzsaki and Wang, 2012; Antonoudiou et al., 2020; Xia et al., 2022). Interestingly, we found that PV+ neurons were regulated bidirectionally in DRN. About 50% of PV+ neurons were putative survival units, and ~20% were putative despair units (Figure 4A). A possible explanation is that PV+ neurons lie in lateral wings of DRN (Li et al., 2020), and GABAergic DRN neuron axons contact with GABAergic neurons in the surrounding brain regions (Barbaresi, 2010). Also, it was reported that stressors such as a swimming test, or a confrontation with an intruder, increase GABA release to 5-HT neurons (Roche et al., 2003). Besides, the waveform cluster analysis showed that putative survival units were wider than despair ones (Figures 4B,C). It suggests the existence of PV+ subtypes in DRN, although this kind of studies is usually performed in the forebrain cortex (Helm et al., 2013; Ghaderi et al., 2018). In other words, there are more complicated neural networks of local inhibitory neurons' subtypes which participated in the balancing regulation in DRN, and thus further exploration needs to be conducted.

In addition, the burst spike patterns were found crucial for putative survival units in the SERT+ neurons (Figure 5B). Although the positive correlation between mice struggling behaviors and burst firing patterns has not been described, early study has confirmed that bursts of 5-HT neuronal action potentials propagate along the axon to the nerve terminal and enhance both the release of 5-HT and its postsynaptic effect (Gartside et al., 2000). Something similar happened in other monoamine neurons as well. It is reported that burst firing elevates extracellular dopamine concentration in an exponential manner (Gonon, 1988; Floresco et al., 2003). Burst firing can be found in various brain regions; however, its functions vary in different regions and cell types (Shao et al., 2021). It was recently reported that the burst firing of lateral habenula neurons was increased in depression animal model (Yang et al., 2018), whereas enhancing burst firing and 5-HT releasing were critical for the regulation of depression and anxiety states in DRN (Hornung, 2003). Although the ionic mechanism of burst firing is different in neuronal subtypes and brain regions (Shao et al., 2021), blockade small-conductance Ca^2+^-activated potassium (SK) channels will promote the bursting firing of 5-HT neurons (Rouchet et al., 2008). In future studies, SK blocker UCL 1,684 should be used to explore its effects on putative survival unit activities. Considering the significant relationship between gamma oscillation/ burst firing fraction in DRN and animal behavior during TST, acutely manipulating gamma oscillation/ burst firing fraction in DRN is necessary to perform in the following studies.

In summary, our results indicated that gamma oscillation and burst fraction were crucial electrophysiological characteristics of TST behavior. Furthermore, PV-positive neurons identified by present data may bidirectionally regulate despair-like behaviors. In addition, this study shown that ~70% of SERT+ neurons in DRN were putative survival units. Electrophysiological characteristic studies of despair-like behavior could provide a new insight into anti-depression drug targets. Meanwhile, it could be concluded that GABAergic interneurons might be a key hub for coding and regulation in local neural network.

## Data Availability Statement

The raw data supporting the conclusions of this article will be made available by the authors, without undue reservation.

## Ethics Statement

The animal study was reviewed and approved by the Ethics Committee of Guangzhou University of Chinese Medicine.

## Author Contributions

LZ, DD, RZ, and YS conceived the research plan. LZ, DD, DL, ZX, and JZ carried out the experiments. LZ, DD, GS, LY, and SB analyzed the results. All authors wrote the manuscript. All authors contributed to the article and approved the submitted version.

## Funding

This work was supported by the National Natural Science Foundation of China (82104557, 82074219, 81873271, and 82104623), Natural Science Foundation of Guangdong Province (2022A1515010230 and 2021A1515012572), China Postdoctoral Science Foundation (2021M700958), and Scientific Research Team Major Project of Guangzhou University of Chinese Medicine (2021xk29).

## Conflict of Interest

The authors declare that the research was conducted in the absence of any commercial or financial relationships that could be construed as a potential conflict of interest.

## Publisher's Note

All claims expressed in this article are solely those of the authors and do not necessarily represent those of their affiliated organizations, or those of the publisher, the editors and the reviewers. Any product that may be evaluated in this article, or claim that may be made by its manufacturer, is not guaranteed or endorsed by the publisher.
